# Association between Dietary Acid Load and Hypertension in Chinese Adults: Analysis of the China Health and Nutrition Survey (2009)

**DOI:** 10.3390/nu15214664

**Published:** 2023-11-03

**Authors:** Feng Lin, Min Zhang, Ruoyu Wang, Meng Sun, Zongfeng Zhang, Yanjiang Qiao, Zhaofeng Zhang

**Affiliations:** 1 School of Chinese Materia Medica, Beijing University of Chinese Medicine, Beijing 102488, China; lfpeptide@126.com; 2 Department of Nutrition & Food Hygiene, School of Public Health, Peking University Health Science Center, Haidian District, Beijing 100191, China; 2111220009@stu.pku.edu.cn (M.Z.); wry7582568@163.com (R.W.); m15910287861@163.com (M.S.); 2011220010@bjmu.edu.cn (Z.Z.); 3 Beijing’s Key Laboratory of Food Safety Toxicology Research and Evaluation, Haidian District, Beijing 100191, China

**Keywords:** dietary acid load, hypertension, potential renal acid load (PRAL), net endogenous acid production (NEAP), blood pressure

## Abstract

(1) Background: Current studies show conflicting results regarding the relationship between dietary acid load (DAL) and blood pressure. (2) Methods: The study used data from the Chinese Health and Nutrition Survey (CHNS) 2009. DAL was assessed on the basis of potential renal acid load (PRAL) and net endogenous acid production (NEAP). To examine the link between DAL and the risk of hypertension, a multivariate logistic regression model was utilized. (3) Results: A total of 7912 subjects were enrolled in the study, of whom 2133 participants had hypertension, a prevalence of 27.0%. After accounting for potential covariates, higher PRAL and NEAP scores were associated with a greater likelihood of developing hypertension, with ORs of 1.34 (95% CI, 1.10–1.62) and 1.29 (95% CI, 1.09–1.53) for PRAL and NEAP scores in Q4, respectively, compared with Q1. In the male group, PRAL and NEAP scores were positively linked to hypertension risk, with ORs of 1.33 (95% CI, 1.06–1.67) and 1.46 (95% CI, 1.14–1.85) for PRAL and NEAP scores in Q4, respectively, compared with Q1, while no significant associations were observed in the female group. Correlations between PRAL scores and hypertension risk lacked significance in the subgroup analyses for participants aged <60 years. There was a significant nonlinear connection observed in the dose–response relationship between DAL (based on PRAL) and hypertension; (4) Conclusions: In Chinese adults, higher PRAL and NEAP scores were positively linked to hypertension risk. This implies that a diet with a low DAL may be a favorable dietary pattern for lowering blood pressure.

## 1. Introduction

Worldwide, hypertension is a crucial risk factor for chronic kidney disease, cardiovascular disease, stroke, and premature death, and it has become an important public health problem affecting human health [1]. The worldwide occurrence of high blood pressure in individuals between the ages of 30 and 79 was 32% for females and 34% for males in 2019, indicating that it is a significant factor in the overall impact of illness worldwide [2]. Although the prevalence of hypertension is generally on the rise, the rate of achieving blood pressure control remains low, with more than 40% of individuals treated for hypertension failing to achieve satisfactory blood pressure control, particularly in low- and middle-income countries [3]. Besides genetic factors, the main causes of hypertension are largely attributable to modifiable environmental factors, among which is a poor diet, which has long been recognized to increase the risk of developing hypertension [4]. Dietary intake of meat, fruit, vegetables, sodium, magnesium, and potassium has an impact on blood pressure values, which can be negative or beneficial. The World Health Organization guidelines for the prevention and treatment of hypertension indicate that dietary changes can reduce blood pressure, and a healthy diet plays a major role in preventing cardiovascular disease [1].

Numerous prior studies have indicated that adopting nutritious eating habits, like the Mediterranean diet and the DASH diet, can decrease the likelihood of hypertension by providing an abundance of anti-inflammatory and antioxidant substances [5,6]. However, in recent years, imbalances in endogenous acid–base homeostasis have been recognized as a driver of many chronic diseases, with potentially significant implications for cardiometabolic risk factors [7]. While food is being digested, it can produce either acid or base precursors (like sulfate or organic anions, such as citrate, malate, etc.), which can have a significant impact on endogenous acid–base homeostasis [8]. Data on food intake can be used to measure DAL and thus estimate the body acid–base changes induced by food intake [9]. It has been suggested that chronic DAL can result in mild metabolic acidosis, potentially leading to elevated cortisol release and impacting blood pressure levels [10]. Frassetto and Remer et al. suggested that DAL could be estimated using mathematical equations, with NEAP and PRAL being the two frequently employed methods for assessment [8,11]. The NEAP score is determined by considering the intake of dietary protein and potassium, while the PRAL score considers the intake of dietary protein, potassium, magnesium, calcium, and phosphorus [12]. Higher PRAL and NEAP scores indicate greater acidogenic potential and correlate with higher dietary acid load [12,13]. Generally, foods that are abundant in protein and phosphorus, primarily those derived from animal sources, like meat, seafood, and cheese, as well as plant sources, like grains, have the potential to increase dietary acid load (DAL) as acidic precursors [14], whereas foods rich in potassium, calcium, and magnesium, such as vegetables and fruits, are prone to alkalizing effects with the exchange of hydrogen ions, which reduce DAL [15].

Recently, many scholars have begun to focus on the correlation between DAL and the likelihood of high blood pressure. However, the existing studies are inconsistent and contradictory. An observational study from Germany involving 6788 subjects showed that elevated DAL scores were linked to higher systolic blood pressure and a greater prevalence of hypertension, with individuals in the highest PRAL score category having a 45% higher likelihood of developing hypertension compared with those in the group with the lowest score group [16]. However, Rotterdam’s study showed no significant association [17]. The majority of the existing proof comes from high-income countries, and the findings of international research might not be relevant to the Chinese population because of variances in geographical regions, ethnicities, and dietary habits. Until now, there has been minimal investigation into this matter in China, with only one cross-sectional study published on a population in southern China, which showed that PRAL was positively associated with hypertension risk among males [18]. However, this study lacked national representation, and the extrapolation of the results was limited. Considering the current high prevalence of hypertension, it is imperative to carry out additional research on the correlation between DAL and the likelihood of developing hypertension to gather more epidemiological proof among the Chinese population and offer scientific recommendations for the prevention and treatment of hypertension. Consequently, we employed the Chinese Health and Nutrition Survey (CHNS) to evaluate whether DAL is linked to the likelihood of hypertension in Chinese adults.

## 2. Materials and Methods

### 2.1. Data Sources and Study Population

This research used open data from the CHNS, a prospective longitudinal study based on household surveys [19]. The first survey was in 1989, and so far, nine rounds of data collection have been conducted, including basic information, disease and health information, socioeconomic status, and dietary and exercise status. Since biological information was only collected in 2009, our study used 2009 data.

**Figure 1:**
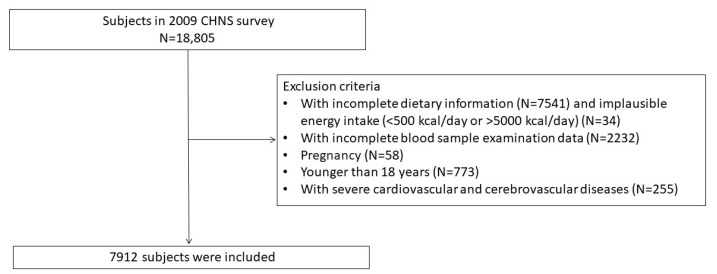
Flowchart outlining the process of selecting participants.

A total of 18,805 subjects participated in the CHNS in 2009. The study ultimately included 7912 participants with complete recorded information on anthropometric indices, lifestyle habits, dietary data, and blood serologic tests. We excluded 7541 subjects without dietary information, 34 participants with unreasonable energy intake, 2232 participants with missing serologic examination data, 58 pregnant women, 773 participants aged <18 years, and 255 participants with severe cardiovascular and cerebrovascular diseases (Figure 1).

A consent form was filled out by each participant before the study. This study was ethically reviewed by the Carolina Population Center at the University of North Carolina at Chapel Hill and the Institute of Nutrition and Health at the Chinese Center for Disease Control and Prevention (CCDC) (2015017).

### 2.2. Evaluation of Diet, PRAL and NEAP

Data on the dietary intake of participants was gathered through a reliable 24 h dietary survey, and dietary consumption information was collected for a total of 3 days (2 weekdays and 1 weekend) [20]. According to the Chinese Food Composition Table (2002 and 2004), daily intakes of energy and nutrients were calculated.

The formulae used to calculate PRAL and NEAP scores were as follows [8,11].

PRAL (mEq/d) = 0.4888 × protein intake (g/d) + 0.0366 × phosphorus (mg/d) − 0.0205 × potassium (mg/d) − 0.0125 × calcium (mg/d) − 0.0263 × magnesium (mg/d)

NEAP (mEq/d) = (54.5 × protein intake (g/d) ÷ potassium intake (mEq/d)) − 10.2.

### 2.3. Definition of Hypertension

Trained physicians used a standard mercury sphygmomanometer to take blood pressure measurements on the subject’s right arm and completed three measurements at 30 s intervals if each measurement was performed normally. Otherwise, subjects were asked to rest for 10–30 min before the next measurement [21]. In our study, the average of three measurements of systolic and diastolic blood pressure was used. Hypertension was defined as an SBP of ≥140 mm Hg and/or a diastolic blood pressure of ≥90 mm Hg, either previously diagnosed by a physician or currently on medication to lower blood pressure [22].

### 2.4. Other Variables

Trained researchers collected sociodemographic information, behavioral lifestyle information, anthropometric indicators, and medical history through the CHNS questionnaire. Sociodemographic information included age (continuous variable), gender, marital status (single, married, or other), region (urban or rural), and education level categorized into 3 levels: low (elementary school and below), medium (middle school, high school, and technical school), and high (college and above). Behavioral lifestyle included smoking status (yes or no), alcohol intake (yes or no), sleep duration (6–9 h, ≤6 h, or ≥9 h), and physical activity (continuous variable). Physical activity was calculated by multiplying subjects’ occupation-related activity, transportation-related activity, and daily physical activity by specific metabolic equivalents (METs) to calculate the corresponding energy metabolism level and then multiplying the MET value of individual activities by the duration of the individual activities to calculate the total metabolic equivalent hours per week. The final unit was MET-hours/week. The calculation of METs was based on the 2011 update of the Physical Activity Compendium and previous studies with data from China [23,24]. Body mass index (BMI, continuous variable) was calculated according to a specific formula based on height (m) and weight (kg). Blood samples were obtained after fasting to measure total cholesterol (TC), low-density lipoprotein cholesterol (LDL), high-density lipoprotein cholesterol (HDL), and triglycerides (TG), and all of the above were statistically analyzed as continuous variables. Hyperuricemia was classified into two groups, “yes” and “no”, on the basis of serum uric acid levels of ≥420 µmol/L (7 mg/dL) in men and ≥360 µmol/L (6 mg/dL) in women [25,26]. Diabetes mellitus was defined as fasting plasma glucose (FPG) ≥7.0 mmol/L, glycosylated hemoglobin ≥6.5%, or self-reported diabetes mellitus and was categorized into “yes” and “no” groups [27]. On the basis of serum creatinine values, the estimated glomerular filtration rate (eGFR) was calculated using the Chronic Kidney Disease Epidemiology Collaboration (CKD-EPI) equation [28].

### 2.5. Statistical Analysis

A descriptive analysis of baseline characteristics was performed using the mean ± standard deviation (SD) or median (interquartile range) for continuous variables and counts (percentages) for categorical variables. In the analysis of variance, Student’s *t*-tests were used for normally distributed continuous variables, Wilcoxon rank-sum tests were used for non-normally distributed variables, and chi-square tests were used for categorical variables.

We used multilevel logistic regression models to explore the relationship between DAL (PRAL and NEAP) and hypertension risk. The odds ratio (OR) was computed by utilizing the quartiles of the DAL scores, with Q1, the group with the lowest scores, serving as the baseline reference group. Since sodium intake affects blood pressure values, it was included as a covariate for statistical analysis. The calculation of sodium in this study included only the contents of sodium in food. Three models were set up in this study to analyze the exposure factors and outcome variables. Model 1 included no covariates; model 2 adjusted for age, sex, education, region, marital status, smoking status, alcohol intake, sleep duration, BMI, TC, TG, HDL-C, LDL-C, hyperuricemia, diabetes mellitus, and eGFR; and model 3 was further adjusted for energy, dietary fiber, and sodium intake. Dividing the population by age (<60 and ≥60 years) and gender, we conducted subgroup analyses. Meanwhile, considering that physical activity can affect blood pressure, the CHNS gathered information on the physical activity of the participants, and physical activity was examined with post hoc analyses because 2937 (37%) of the study subjects were missing data on physical activity in our study. After excluding subjects with missing data, physical activity was used as a covariate to investigate the potential impact of this covariate with model 3. Taking into account that subjects may have changed their existing diets because of disease conditions, we also performed sensitivity analyses, excluding participants with diabetes and an eGFR of <60 mL/min/1.73 m^2^.

To investigate the dose–response correlation between DAL and hypertension prevalence, we examined the nonlinear relationship using a multivariate-adjusted restricted cubic spline (model 3).

A statistically significant value was considered when *p* < 0.05 on both sides. Statistical analysis was performed on all data using SPSS 23. The figures were generated by GraphPad Prism 9.4.1 and R version 4.2.2.

## 3. Results

### 3.1. Participants Characteristics

Our study enrolled 7912 subjects with a mean age of 50.2 ± 14.9 years, of whom 4187 (52.9%) were females and 3725 (47.1%) were males. The study included 2133 individuals with hypertension, indicating a hypertension prevalence rate of 27.0%. A total of 5496 participants (69.5%) lived in rural areas, 5367 (67.8%) had a sleep duration of 6–9 h, 2450 (31.0%) and 1678 (21.2%) history of smoking and alcohol intake, respectively, 1195 (15.1%) had hyperuricemia, and 827 (10.5%) had diabetes mellitus. The median PRAL and NEAP were 24.7 mEq/d and 76.6 mEq/d, respectively. Subjects were divided into two groups according to whether they had hypertension or not, and the distribution of their characteristics is described in Table 1. The hypertension group was older and had higher TC, TG, LDL-C, and BMI and lower eGFR and physical activity levels compared with the non-hypertension group.

The nutrient intake of the subjects was expressed using energy adjustment, as shown in Table 2. The hypertension group consumed a higher amount of energy-adjusted total protein, plant protein, calcium, phosphorus, and sodium, and the non-hypertension group demonstrated more energy intake. There were no differences in energy-adjusted carbohydrate, fat, dietary fiber, animal protein, potassium, or magnesium consumption between the two groups.

### 3.2. The Correlation between DAL and Hypertension

#### 3.2.1. PRAL and Hypertension

As shown in Table 3, in the logistic regression statistics of model 1, we found no correlation between the PRAL score and hypertension risk. The reference group comprised the data of the first quartile (Q1), and the OR values of Q2, Q3, and Q4 were 0.92 (0.81–1.07), 0.88 (0.76–1.01), and 1.03 (0.90–1.19), respectively, which were not statistically different. By contrast, in model 2 and model 3, there was a correlation between elevated PRAL scores and an increased risk of hypertension. Refer to Table S1 for complete information. When PRAL was used as a continuous variable in model 3, positive correlations were also found; these results are not shown.

#### 3.2.2. NEAP and Hyperuricemia

Consistent with the PRAL statistics, the NEAP scores and the risk of hypertension were not statistically significant in model 1. However, after further adjustment for covariates in model 2 and model 3, the findings indicated that increased NEAP scores were linked to an elevated risk of hypertension (Table 4). Model 3 showed ORs of 1.09 (0.93–1.28), 1.12 (1.03–1.43), and 1.29 (1.09–1.53) in Q2, Q3, and Q4, respectively, compared with Q1. The detailed results are shown in Table S2. Significant positive correlations also existed when NEAP was used as a continuous variable in model 3; these results are not shown.

#### 3.2.3. Stratification Analysis Based on Gender and Age

**Figure 2:**
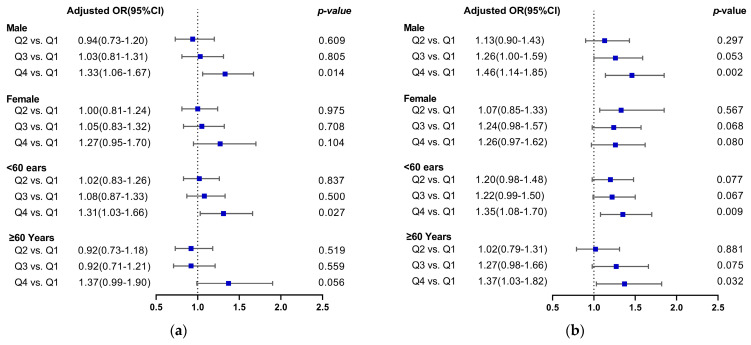
Forest plot of DAL versus hypertension risk after age and sex stratification. (**a**) PRAL and the risk of hypertension; (**b**) NEAP and the risk of hypertension. The blue squares represent multivariate-adjusted odds ratio.

In the female subgroup, there was no correlation between PRAL or NEAP scores and a higher likelihood of developing hypertension. In the male group, after controlling for confounding variables in model 3, there was an increased risk of hypertension in Q4 compared with Q1 for both the PRAL and NEAP scores, with ORs of 1.33 (1.06–1.67), and 1.46 (1.14–1.85), respectively. Subgroup analysis based on age grouping showed that elevated PRAL was associated with an increased risk of hypertension in participants aged <60 years, with an OR of 1.31 (1.03–1.66) in Q4 compared with Q1, which was a statistically significant difference. However, in participants aged ≥60 years, there was no correlation between the above two after correcting for potential confounders. In participants aged <60 years group and ≥60 years, higher NEAP scores were significantly and positively correlated with a higher hypertension prevalence, with ORs of 1.35 (1.08–1.70) and 1.37 (1.03–1.82) in Q4, respectively, as shown in Figure 2a,b.

### 3.3. Sensitivity Analysis

To determine the stability of the results, we performed sensitivity analyses. Considering that exercise and physical activity can affect blood pressure, questionnaires were used to determine subjects’ exercise and physical activity in the CHNS; however, physical activity data were missing for 2937 study subjects (37%) in our study, so after excluding subjects with missing data, physical activity was included as a covariate, and the potential effect of this variable was further investigated with model 3. The findings of the research were in line with the outcomes of excluding physical activity. In addition, when the sensitivity analyses excluded subjects with diabetes mellitus and an eGFR of <60 mL/min/1.73 m^2^, the correlation between PRAL, NEAP, and hypertension remained significant, and our findings were not altered (Tables S3–S5).

### 3.4. DAL and Hypertension Risk Based on Restricted Cubic Spline Analysis (RCS)

**Figure 3:**
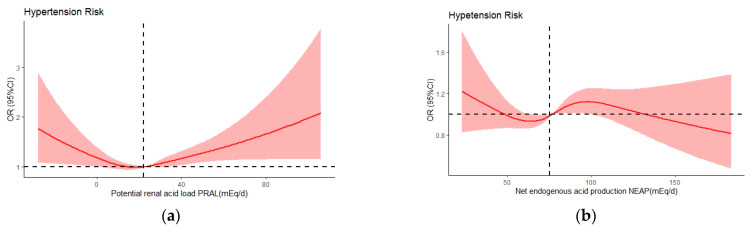
Odds ratios (ORs) of PRAL (**a**) and NEAP (**b**) versus hypertension risk adjusted by model 3. The red lines indicate multivariate-adjusted odds ratio and the red shadows indicate the 95%CIs derived from restricted cubic splineregression.The median intakes were set as references (black dashed lines) (OR = 1.00). CI, confidence limit.

The analysis using the model 3-adjusted restricted cubic spline demonstrated a noteworthy nonlinear correlation between PRAL and hypertension risk (P for nonlinear trend = 0.0037), with an overall U-shaped relationship, as shown in Figure 3a. For NEAP, a significant nonlinear association was not observed (P for nonlinear trend = 0.0827), as depicted in Figure 3b.

## 4. Discussion

This is the first large cross-sectional study on the association between DAL and hypertension in Chinese adults that is geographically diverse, contains extensive data, and corrects for many potential confounders. When multiple covariates were adjusted in model 3, our study discovered that dietary acid load levels, as evaluated by PRAL and NEAP, were linked to the prevalence of hypertension, with higher scores associated with a higher risk of hypertension. This positive association was statistically significant among males and participants aged <60 years; moreover, the positive association between NEAP and hypertension persisted in participants aged ≥60 years. The sensitivity analyses yield consistent results with the statistical findings of the overall population.

Regarding the correlation between DAL and the likelihood of high blood pressure, our findings align with the outcomes of multiple prior observational and prospective investigations. A study on a large representative sample of Japanese adults revealed that higher PRAL and NEAP scores were positively linked to higher systolic and diastolic blood pressure in males. Conversely, in females, only systolic blood pressure showed a positive association with PRAL and NEAP scores, while diastolic blood pressure did not. Importantly, these results were unaffected by the influence of BMI on blood pressure [29]. According to a recent systematic review and meta-analysis, adult populations with higher DAL scores have a correspondingly increased risk of hypertension. A high PRAL is associated with a 14% increase in the risk of hypertension, while a high NEAP is associated with a 35% increase [30]. In a study conducted in the United States, 87,293 women with no prior hypertension were examined. The findings revealed a significant association between NEAP and the development of hypertension, with a 14% increased risk of hypertension in the group with the highest scores compared with the group with the lowest scores (HR: 1.14; 95% CI: 1.05, 1.24) after adjusting for the effects of potential covariates [31]. However, in our subgroup analyses according to gender, we did not find consistent results. Specifically, we found a positive association between PRAL and NEAP and hypertension risk in males, whereas no significant correlation was detected in females. In a cross-sectional study in China’s Guangdong Province, a higher PRAL was found to be associated with hypertension risk in the male population, but this association was not statistically significant in the female group. Additionally, there was no association between NEAP and hypertension risk in males or females [18]. A meta-analysis of DAL and hypertension risk found conflicting results for gender-stratified analyses, and further validation is needed for the combined effects across genders [30]. The different findings considered may be related to the research design and subject exclusion and inclusion criteria, or they may have resulted from differences in geographic characteristics, genetic factors, behavioral habits, and diet quality. In addition, studies have reported that some biological factors in females are protective, such as estrogen [32]. This study offers epidemiological evidence to examine whether there are disparities between genders in the occurrence of DAL and hypertension risk among the Chinese population.

In subgroup analyses based on age grouping, we found no correlation between PRAL scores and risk of hypertension in those aged ≥60 years. Results from both cross-sectional and longitudinal studies in older populations from Sweden have shown no correlations between DAL assessed using PRAL and NEAP and various blood pressure indices, which is consistent with our findings on PRAL [33]. The Rotterdam Study involved participants with an average age of 65 years, which did not find any proof to back the claim that PRAL, NEAP, and the likelihood of hypertension in elderly individuals are connected [17]. Nevertheless, in our research, Q4 of NEAP was found to have a greater risk of developing hypertension than Q1 in people aged ≥60 years. This discrepancy should be interpreted with caution given the different calculation formulas for estimating dietary acid load for PRAL and NEAP. The PRAL calculation includes other nutrients in addition to dietary protein intake and potassium, taking into account the ionic balance of magnesium and calcium as well as the dissociation of phosphate, whereas the NEAP calculation assumes that all positive minerals can be ignored except potassium, and it considers only dietary protein and potassium involvement, which may affect accuracy [34]. In addition, Remer et al. indicated that compared with NEAP, PRAL exhibited a stronger correlation with net acid excretion in 24 h urine [11]. Currently, there is a lack of clarity on the reasons why no association has been found between DAL and hypertension in the elderly population. Research indicates that hypertension in elderly individuals may be primarily attributable to other causes, such as atherosclerosis, attenuating the effect of dietary factors, such as DAL, on blood pressure [35]. In addition, renal function affects endogenous acid elimination with a corresponding decline in eGFR with increasing age, leading to the limitation of acid elimination, and therefore, older adults may be better able to tolerate diets with a high acid-forming potential [17].

The underlying mechanisms of the association between DAL and the risk of hypertension are not clearly defined, but several studies have proposed reasonable mechanisms to explain the positive association between them. First, low-grade metabolic acidosis due to diet can increase the proton load and decreases blood pH, and the body’s acid–base homeostasis affects renal absorption of calcium and magnesium [36]. Some research evidence suggests that a high acid load can cause an increase in calcium and magnesium excretion and altered calcium homeostasis, which is associated with elevated blood pressure [37,38]. Second, low levels of metabolic acids can stimulate increased cortisol secretion and decreased inactivation, which can lead to increased blood pressure [10]. Third, the effect of DAL on renal function may also indirectly affect blood pressure. Studies have demonstrated that an elevated DAL can lead to a rise in hydrogen ion concentration in the renal tubules, along with an increase in angiotensin II and aldosterone. This slight acidosis prompts the generation of ammonia by tubular cells, which is utilized to counterbalance the hydrogen ion burden. A prolonged elevation of ammonia concentration in the kidneys can have adverse effects on renal well-being, potentially impacting blood pressure in the kidneys and the renin–angiotensin–aldosterone system [39]. In the analysis of our subgroups, the inclusion of eGFR in the adjustments did not weaken the positive associations between NEAP, PRAL, and the occurrence of hypertension in males and participants aged <60 years. Finally, fruits and vegetables, which are characterized by a low DAL, contain ample amounts of potassium, magnesium, and calcium and their intake has a negative correlation with the likelihood of developing hypertension [40]. Meanwhile, a diet with a low DAL is rich in dietary fiber, which facilitates the cultivation of diverse intestinal microbiota, leading to the promotion of intestinal ecological balance, which may have a positive effect on blood pressure [41,42].

Studies have demonstrated that a higher dietary sodium intake is positively associated with the risk of hypertension [43] and that dietary sodium intake can be measured by a variety of methods, including dietary and urinary assessments [44]. The 24 h urinary sodium excretion test is considered the gold standard for determining dietary sodium. However, it has been suggested that daily dietary sodium intake fluctuates and that the best way to assess sodium intake is to collect multiple complete 24 h urine samples during nonconsecutive 3- to 10-day periods to reflect sodium excretion [45]; the complexity of such measurements is more difficult to generalize and apply in large sample population surveys. Dietary assessment methods (3-day, 24-h, and food frequency methods) can provide valid estimates of dietary nutrient intake and are recommended for assessing dietary nutrient intake [46,47]. A meta-analysis by McLean et al. found that the 24 h dietary recall method tended to underestimate dietary sodium intake compared with the 24 h urinary sodium excretion [48]. Now, more studies have begun to use dietary sodium as a substitute for urinary sodium to assess the relationship between sodium intake and disease, especially some large studies. A study using data from the National Health and Nutrition Examination Survey (NHANES) showed that excessive dietary sodium intake was associated with high systolic blood pressure [49]. A meta-analysis of dietary sodium and cardiovascular disease risk examining both 24 h urinary sodium assessments and dietary questionnaires assessing sodium intake showed that high sodium intake is an important risk factor for cardiovascular disease, with a 6% increase in cardiovascular disease risk for each additional 1 g of sodium in the diet [50]. We used data from a large-sample study conducted in a wide geographical area of China in which sodium intake was calculated by a 3-day 24 h dietary survey, and it has been shown that the use of multiple 24 h dietary recalls can help to narrow the discrepancy between dietary sodium assessments and 24 h urinary sodium assessments [48]. Therefore, even though the CHNS did not collect indicators for urinary sodium assessment, we still consider the results of the study to be representative, valuable, and interesting. In our study, sodium was adjusted as a covariate, and we were more concerned about the effect of DAL on blood pressure. In an investigative study in Japan on DAL and hypertension that adjusted for dietary sodium as a covariate, a high DAL was found to be associated with an increased prevalence of hypertension [51], which is consistent with our findings. In future studies, we can further explore the relationship between DAL and the risk of hypertension by using urinary sodium excretion as a covariate. There were certain advantages in the present investigation. First, we employed various parameters to define hypertension in our study. Besides diagnosing hypertension by averaging three repeated measurements of blood pressure, we also included subjects who had been previously diagnosed by a physician, were taking antihypertensive medication, and self-reported their diagnosis, which allowed for a more accurate reflection of the reality of an individual’s blood pressure. Second, we used a large geographically representative sample and controlled for a wide range of potentially confounding variables to examine the relationship between DAL and the prevalence of hypertension, and the findings were reliable and representative. The findings of our study provide supporting evidence for investigating the correlation between DAL and the likelihood of developing hypertension in Chinese populations, and theyindicate that adopting a diet with a low DAL may serve as an efficient dietary approach to managing and decreasing hypertension risk.

It is also necessary to discuss the limitations of this study. First, the exploration of the relationship between DAL and hypertension in the present study utilized a cross-sectional design, and the association findings do not represent a causal relationship. Longitudinal studies can be conducted at a later stage to further validate these findings. Second, the calculation of PRAL and NEAP scores relied on mathematical modeling formulas rather than objective measurement assessments, which may not have been integrated for the digestion and absorption of nutrients caused by individual differences [34]. However, it is not easy to measure the acid load of urine in large studies. The PRAL and NEAP scores have been validated and are frequently utilized in epidemiological research, displaying a strong association with acid load as measured in 24 h urine [8,11]. Finally, the CHNS did not collect data reflecting low-grade organic metabolic acidosis, such as serum pH, urine pH, or bicarbonate concentration, but some studies conducted in healthy and CKD populations have shown that DAL correlates well with endogenous acid–base status [14,52,53].

## 5. Conclusions

To sum up, we discovered a positive correlation between elevated PRAL and hypertension risk in males and participants aged <60 years, and these associations were not statistically significant in females or participants aged ≥60 years. In relation to the connection between NEAP and hypertension, significant associations were found in males, participants aged <60 years, and participants aged ≥60 years. There was a significant nonlinear association in the dose–response relationship between DAL (based on PRAL) and hypertension. Consuming diets that have higher PRAL or NEAP scores could potentially increase the risk of hypertension, while following a low-DAL dietary pattern may prove to be highly beneficial in reducing blood pressure.

## Figures and Tables

**Table 1:** Characteristics of subjects categorized by their hypertension status.

Variable	Overall (*n* = 7412)	Hypertension (*n* = 2133)	Non-Hypertension (*n* = 5779)	*p*-Value
Age (years)	50.2 (14.9)	58.7 (12.9)	47.1 (14.3)	<0.0001
Gender				
Male	3725 (47.1%)	1058 (49.6%)	2667 (46.1%)	<0.01
Female	4187 (52.9%)	1075 (50.4%)	3112 (53.9%)	
PRAL ^1^ group	24.7 (17.3)	24.9 (18.1)	24.6 (16.9)	0.532
NEAP ^2^ group	76.6 (22.9)	77.4 (23.1)	76.4 (22.8)	0.085
Marital status				
Single	489 (6.2%)	38 (1.8%)	451 (7.8%)	<0.0001
Married	6677 (84.4%)	1770 (83%)	4907 (84.9%)	
Other	746 (9.4%)	325 (15.2%)	421 (7.3%)	
Region				
Urban	2416 (30.5%)	660 (30.9%)	1756 (30.4%)	0.633
Rural	5496 (69.5%)	1473 (69.1%)	4023 (69.6%)	
Education level				
Low	3460 (43.7%)	1192 (55.9%)	2268 (39.2%)	<0.0001
Middle	4088 (51.7%)	888 (41.6%)	3200 (55.4%)	
High	364 (4.6%)	53 (2.5%)	311 (5.4%)	
Smoking status				
No	5462 (69.0%)	1426 (66.9%)	4036 (69.8%)	<0.05
Yes	2450 (31.0%)	707 (33.1%)	1743 (30.2%)	
Alcohol intake				
No	6234 (78.8%)	1621 (76%)	4613 (79.8%)	<0.0001
Yes	1678 (21.2%)	512 (24%)	1166 (20.2%)	
Physical activity ^3^ (MET·hours/week)	131.00 (70.63–239.43)	123.83 (53.33–227.33)	133.08 (73.67–243.92)	<0.0001
Hyperuricemia				
Yes	1195 (15.1%)	477 (22.4%)	718 (12.4%)	<0.0001
No	6717 (84.9%)	1656 (77.6%)	5061 (87.6%)	
Diabetes				
Yes	827 (10.5%)	401 (18.8%)	426 (7.4%)	<0.0001
No	7085 (89.5%)	1732 (81.2%)	5353 (92.6%)	
TC ^4^ (mmol/L)	4.86 (1.00)	5.10 (1.02)	4.76 (0.98)	<0.0001
TG ^5^ (mmol/L)	1.65 (1.44)	1.93 (1.57)	1.55 (1.38)	<0.0001
LDL-C ^6^ (mmol/L)	2.97 (0.98)	3.16 (1.03)	2.90 (0.96)	<0.0001
HDL_C ^7^ (mmol/L)	1.44 (0.50)	1.42 (0.62)	1.45 (0.45)	0.065
eGFR ^8^ (mL/min/1.73m^2^)	79.6 (16.7)	72.7 (16.4)	82.2 (16.1)	<0.0001
BMI ^9^ (kg/m^2^)	23.3 (3.4)	24.6 (3.7)	22.8 (3.2)	<0.0001
Sleep duration (hours)				
6–9	5367 (67.8%)	1371 (64.3%)	3996 (69.2%)	<0.0001
≤6	756 (9.6%)	276 (12.9%)	480 (8.3%)	
≥9	1789 (22.6%)	486 (22.8%)	1303 (22.5%)	

^1^ PRAL: potential renal acid load; ^2^ NEAP: net endogenous acid production; ^3^ data were available for 4975 subjects due to 2937 missing dates on physical activity; ^4^ TC: total cholesterol; ^5^ TG: triglycerides; ^6^ LDL-C: low-density lipoprotein cholesterol; ^7^ HDL-C: high-density lipoprotein cholesterol; ^8^ eGFR: estimated glomerular filtration rate; ^9^ BMI: body mass index.

**Table 2:** Intakes of energy and nutrients (energy-adjusted, per 1000 kcal).

Nutrients	Hypertension	Non-Hypertension	*p*-Value
Energy (Kcal)	1836.29 (1450.32–2300.57)	1897.18 (1521.66–2335.36)	<0.0001
Carbohydrate (g/1000 kcal)	164.84 (143.12–187.41)	166.23 (145.15–188.09)	0.179
Protein (g/1000 kcal)	39.22 (33.79–47.32)	38.42 (33.05–46.21)	<0.005
Animal protein (g/1000 kcal)	13.98 (6.53–24.28)	14.35 (6.73–23.95)	0.615
Plant protein (g/1000 kcal)	22.92 (18.77–27.80)	22.23 (18.37–27.09)	<0.005
Fat (g/1000 kcal)	22.26 (14.02–30.10)	22.10 (14.11–29.66)	0.539
Cholesterol (mg/1000 kcal)	172.31 (82.71–283.74)	167.66 (86.42–266.41)	0.41
Dietary fiber (g/1000 kcal)	5.83 (4.34–7.78)	5.66 (4.31–7.54)	0.112
Calcium (mg/1000 kcal)	220.49 (164.43–298.55)	206.77 (156.41–277.70)	<0.0001
Phosphorous (mg/1000 kcal)	570.27 (502.62–651.95)	558.27 (492.14–636.08)	<0.0001
Potassium (mg/1000 kcal)	996.53 (831.31–1219.34)	996.24 (831.93–1210.87)	0.744
Magnesium (mg/1000 kcal)	162.34 (140.08–189.20)	160.96 (138.85–186.66)	0.09
Sodium (mg/1000 kcal)	368.13 (212.67–591.92)	333.92 (201.38–591.92)	<0.005

**Table 3:** ORs (and 95%CIs) based on PRAL for hypertension risk.

PRAL	Model 1		Model 2		Model 3	
	OR (95% CI)	*p*	OR (95% CI)	*p*	OR (95% CI)	*p*
Q1	1 (ref)		1 (ref)		1 (ref)	
Q2	0.92 (0.81–1.07)	0.283	0.95 (0.81–1.11)	0.481	0.97 (0.83–1.14)	0.725
Q3	0.88 (0.76–1.01)	0.072	0.99 (0.84–1.15)	0.849	1.03 (0.87–1.22)	0.727
Q4	1.03 (0.90–1.19)	0.646	1.24 (1.06–1.46)	<0.01	1.34 (1.10–1.62)	<0.01

**Table 4:** ORs (and 95%CIs) based on NEAP for hypertension risk.

NEAP	Model 1		Model 2		Model 3	
	OR (95% CI)	*p*	OR (95% CI)	*p*	OR (95% CI)	*p*
Q1	1 (ref)	0.001	1 (ref)		1 (ref)	
Q2	0.92 (0.81–1.07)	0.283	1.07 (0.91–1.25)	0.423	1.09 (0.93–1.28)	0.303
Q3	0.88 (0.76–1.01)	0.072	1.17 (1.00–1.37)	0.051	1.12 (1.03–1.43)	<0.05
Q4	1.03 (0.90–1.19)	0.646	1.23 (1.05–1.44)	<0.05	1.29 (1.09–1.53)	<0.005

## Data Availability

Publicly available datasets were analyzed in this study. These data can be found here: https://www.cpc.unc.edu/projects/china/data (accessed on 10 December 2022).

## Supplementary Materials

The following supporting information can be downloaded at: https://www.mdpi.com/article/10.3390/nu15214664/s1, Table S1: ORs (and 95% CIs) based on PRAL for hypertension risk; Table S2: ORs (and 95% CIs) based on NEAP for hypertension risk; Table S3: ORs (and 95% CIs) of PRAL, NEAP, and hypertension risk in subjects with physical activity deficit exclusion (adjusted with model 3); Table S4: ORs (and 95% CIs) of PRAL, NEAP, and hypertension risk in nondiabetic subjects (adjusted with model 3); Table S5: ORs (and 95% CIs) of PRAL, NEAP, and hypertension risk in subjects with eGFR ≥ 60 mL/min/1.73 m^2^ (adjusted with model 3).
