# Supplementary material for: Association between Dietary Acid Load and Hypertension in Chinese Adults: Analysis of the China Health and Nutrition Survey (2009)
Source: Nutrients. 2023 Nov 3;15(21):4664. doi: 10.3390/nu15214664 (PMC10647800; doi:10.3390/nu15214664)
Supplement: Supplementary file 1 [file nutrients-15-04664-s001.zip › nutrients-2656544-supplementary.pdf]

Supplementary Materials

Table S1: ORs (and 95%CIs) based on PRAL for hypertension risk.

	**Model 1**	***p***	**Model 2**	***p***	**Model 3**	***p***
Age			1.06（1.05-1.07）	<0.001	1.06（1.05-1.07）	<0.001
Gender						
Male			1(ref)		1(ref)	
Female			0.84(0.73-0.95)	<0.01	0.83(0.72-0.94)	<0.01
PRAL						
Q1	1(ref)		1(ref)		1(ref)	
Q2	0.92(0.81-1.07)	0.283	0.95(0.81-1.11)	0.481	0.97(0.83-1.14)	0.725
Q3	0.88(0.76-1.01)	0.072	0.99(0.84-1.15)	0.849	1.03(0.87-1.22)	0.727
Q4	1.03(0.90-1.19)	0.646	1.24(1.06-1.46)	<0.01	1.34(1.10-1.62)	<0.01
Marital status						
Single			1(ref)		1(ref)	
Married			0.83(0.56-1.21)	0.333	0.85(0.58-1.24)	0.392
Other			0.89(0.58-1.)37	0.588	0.92(0.60-1.41）	0.696
Region						
Urban			1(ref)		1(ref)	
Rural			1.09(0.96-1.23)	0.187	1.13(0.99-1.28)	0.062
Education						
Low			1(ref)		1(ref)	
Middle			0.93(0.82-1.06)	0.286	0.93(0.82-1.06)	0.276
High			0.53(0.38-0.74)	<0.001	0.53(0.38-0.74)	<0.001
Smoking status						
No			1(ref)		1(ref)	
Yes			1.06(0.91-1.23)	0.47	1.06(0.91-1.23)	0.454
Alcohol intake						
No			1(ref)		1(ref)	
Yes			1.14(0.99-1.33)	0.074	1.14(0.98-1.32)	0.083
Hyperuricemia						
No			1(ref)		1(ref)	
Yes			1.23(1.05-1.45)	<0.05	1.25(1.06-1.47)	<0.05
Diabetes						
No			1(ref)		1(ref)	
Yes			1.44(1.22-1.70)	<0.001	1.43(1.21-1.69)	<0.001
TC			0.97(0.85-1.10)	0.587	0.97(0.85-1.10)	0.604
TG			1.07(1.02-1.12)	<0.005	1.07(1.03-1.12)	<0.005
LDL-C			1.07(1.02-1.14)	<0.05	1.07(1.01-1.13)	<0.05
HDL_C			1.05(0.94-1.17)	0.394	1.06(0.94-1.18)	0.347
eGFR（ml/min/1.73m^2）			0.99(0.98-0.99)	<0.05	0.99(0.98-0.99)	<0.05
BMI（kg/m^2）			1.16(1.14-1.18)	<0.001	1.16(1.14-1.18)	<0.001
Sleep duration(hours)						
6-9			1(ref)		1(ref)	

≤6			1.11(0.93-1.33)	0.248	1.09(0.91-1.30)	0.378
≥9			0.96(0.84-1.10)	0.589	0.96(0.84-1.10)	0.579
Energy (Kcal)					1.00(1.00-1.01)	0.128
Sodium, per 1SD					1.09(1.02-1.16)	<0.05
Dietary fiber						
Q1					1(ref)	
Q2					0.96(0.83-1.10)	0.528
Q3					1.15(0.98-1.35)	0.097

PRAL: potential renal acid load; TC: total cholesterol; TG: triglycerides; LDL-C: low-density lipoprotein cholesterol; HDL-C: high-density lipoprotein cholesterol; BMI: body mass index; eGFR: estimated glomerular filtration rate.

Table S2: ORs (and 95%CIs) based on NEAP for hypertension risk.

	Model 1	*p*	Model 2	*p*	Model 3	*p*
Age			1.06（1.05-1.06）	<0.001	1.06(1.05-1.07)	<0.001
Gender						
Male			1(ref)		1(ref)	
Female			0.83(0.72-0.94)	<0.005	0.84(0.73-0.95)	<0.01
NEAP						
Q1	1(ref)		1(ref)		1(ref)	
Q2	0.92(0.81-1.07)	0.283	1.07(0.91-1.25)	0.423	1.09(0.93-1.28)	0.303
Q3	0.88(0.76-1.01)	0.072	1.17(1.00-1.37)	0.051	1.12(1.03-1.43)	<0.05
Q4	1.03(0.90-1.19)	0.646	1.23(1.05-1.44)	<0.05	1.29(1.09-1.53)	<0.005
Marital status						
Single			1(ref)		1(ref)	
Married			0.84(0.57-1.23)	0.369	0.84(0.58-1.24)	0.385
Other			0.90(0.59-1.39)	0.636	0.92(0.60-1.41)	0.693
Region						
Urban			1(ref)		1(ref)	
Rural			1.09(0.96-1.23)	0.203	1.10(0.97-1.25)	0.131
Education						
Low			1(ref)		1(ref)	
Middle			0.93(0.82-1.06)	0.274	0.92(0.81-1.04)	0.19
High			0.53(0.38-0.74)	<0.001	0.52(0.37-0.73)	<0.001
Smoking status						
No			1(ref)		1(ref)	
Yes			1.06(0.91-1.23)	0.471	1.06(0.91-1.24)	0.435
Alcohol intake						
No			1(ref)		1(ref)	
Yes			1.15(0.99-1.33)	0.061	1.14(0.98-1.32)	0.085
Hyperuricemia						
No			1(ref)		1(ref)	
Yes			1.24(1.05-1.46)	<0.05	1.24(1.06-1.46)	<0.01
Diabetes						

No	1(ref)		1(ref)	
Yes	1.43(1.21-1.69)	<0.001	1.43(1.20-1.68)	<0.001
TC	0.97(0.85-1.10)	0.58	0.97(0.85-1.10)	0.626
TG	1.07(1.03-1.11)	<0.005	1.07(1.03-1.12)	<0.005
LDL-C	1.07(1.01-1.14)	<0.05	1.07(1.01-1.14)	<0.05
HDL_C	1.05(0.94-1.17)	0.381	1.05(0.94-1.18)	0.351
eGFR（ml/min/1.73m^2）	0.99(0.98-0.99)	<0.05	0.99(0.98-0.99)	<0.05
BMI（kg/m2）	1.17(1.14-1.19)	<0.001	1.16(1.14-1.18)	<0.001
Sleep duration(hours)				
6-9	1(ref)		1(ref)	
≤6	1.11(0.93-1.33)	0.239	1.09(0.91-1.31)	0.346
≥9	0.96(0.83-1.10)	0.527	0.96(0.83-1.10)	0.538
Energy(Kcal)			1.00(1.00-1.01)	0.18
Sodium, per 1SD			1.07(1.01-1.14)	0.04
Dietary fiber				
Q1			1(ref)	
Q2			0.97(0.84-1.12)	0.698
Q3			1.18(1.01-1.37)	<0.05

NEAP: net endogenous acid production; TC: total cholesterol; TG: triglycerides; LDL-C: low-density lipoprotein cholesterol; HDL-C: high-density lipoprotein cholesterol; BMI: body mass index; eGFR: estimated glomerular filtration rate.

Table S3: ORs (and 95%CIs) of PRAL, NEAP and hypertension risk in subjects with physical activity deficit exclusion (adjusted with model 3).

PRAL	Model 3		NEAP	Model 3	
	**OR (95%CI)**	***p***		**OR (95%CI)**	***p***
Q1	1(ref)		Q1	1(ref)	
Q2	1.00(0.81-1.24)	0.982	Q2	1.22(0.98-1.51)	0.074
Q3	1.02(0.83-1.26)	0.858	Q3	1.22(0.98-1.53)	0.077
Q4	1.32(1.07-1.63)	0.009	Q4	1.33(1.04-1.68)	0.021

PRAL: potential renal acid load; NEAP: net endogenous acid production.

Table S4: ORs (and 95%CIs) of PRAL, NEAP and hypertension risk in non-diabetic subjects (adjusted with model 3).

PRAL	Model 3		NEAP	Model 3	
	**OR (95%CI)**	***p***		**OR (95%CI)**	***p***
Q1	1(ref)		Q1	1(ref)	
Q2	0.97(0.82-1.15)	0.751	Q2	1.07(0.90-1.27)	0.458
Q3	1.01(0.85-1.20)	0.939	Q3	1.23(1.03-1.46)	0.022
Q4	1.29(1.09-1.53)	0.004	Q4	1.35(1.12-1.61)	0.001

PRAL: potential renal acid load; NEAP: net endogenous acid production.

Table S5: ORs (and 95%CIs) of PRAL, NEAP and hypertension risk in subjects with eGFR⩾ 60 ml/min/1.73m^2 (adjusted with model 3).

PRAL	Model 3		NEAP	Model 3	
	**OR (95%CI)**	***p***		**OR (95%CI)**	***p***
Q1	1(ref)		Q1	1(ref)	
Q2	0.99(0.83-1.18)	0.925	Q2	1.09(0.92-1.30)	0.330
Q3	1.12(0.93-1.34)	0.222	Q3	1.25(1.05-1.48)	0.014
Q4	1.41(1.14-1.74)	0.001	Q4	1.34(1.12-1.61)	0.002

PRAL: potential renal acid load; NEAP: net endogenous acid production; eGFR: estimated glomerular filtration rate.
